# A Review of Injectable Polymeric Hydrogel Systems for Application in Bone Tissue Engineering

**DOI:** 10.3390/molecules21111580

**Published:** 2016-11-21

**Authors:** Pariksha J. Kondiah, Yahya E. Choonara, Pierre P. D. Kondiah, Thashree Marimuthu, Pradeep Kumar, Lisa C. du Toit, Viness Pillay

**Affiliations:** Wits Advanced Drug Delivery Platform Research Unit, Department of Pharmacy and Pharmacology, School of Therapeutic Sciences, Faculty of Health Sciences, University of the Witwatersrand, Johannesburg, 7 York Road, Parktown 2193, South Africa; 549348@students.wits.ac.za (P.J.K.); yahya.choonara@wits.ac.za (Y.E.C.); pierre.kondiah@wits.ac.za (P.P.D.K.); thashree.marimuthu@wits.ac.za (T.M.); pradeep.kumar@wits.ac.za (P.K.); lisa.dutoit@wits.ac.za (L.C.d.T.)

**Keywords:** biodegradable, thermo-responsive polymers, hydrogels, tissue engineering, drug delivery, stimuli-responsive

## Abstract

Biodegradable, stimuli-responsive polymers are essential platforms in the field of drug delivery and injectable biomaterials for application of bone tissue engineering. Various thermo-responsive hydrogels display water-based homogenous properties to encapsulate, manipulate and transfer its contents to the surrounding tissue, in the least invasive manner. The success of bioengineered injectable tissue modified delivery systems depends significantly on their chemical, physical and biological properties. Irrespective of shape and defect geometry, injectable therapy has an unparalleled advantage in which intricate therapy sites can be effortlessly targeted with minimally invasive procedures. Using material testing, it was found that properties of stimuli-responsive hydrogel systems enhance cellular responses and cell distribution at any site prior to the transitional phase leading to gelation. The substantially hydrated nature allows significant simulation of the extracellular matrix (ECM), due to its similar structural properties. Significant current research strategies have been identified and reported to date by various institutions, with particular attention to thermo-responsive hydrogel delivery systems, and their pertinent focus for bone tissue engineering. Research on future perspective studies which have been proposed for evaluation, have also been reported in this review, directing considerable attention to the modification of delivering natural and synthetic polymers, to improve their biocompatibility and mechanical properties.

## 1. Introduction

**Figure 1:**
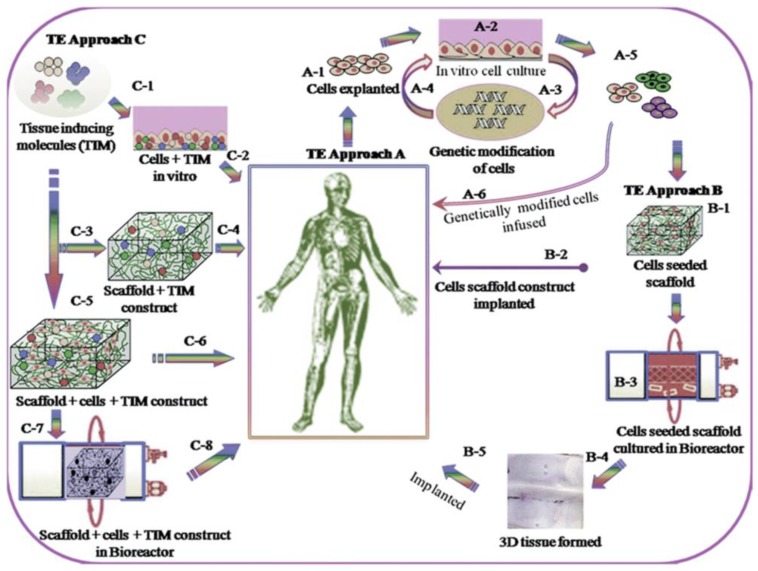
Schematic representing the outlined procedure undertaken for tissue engineering and drug delivery (adapted with permission from Khan et al., 2015).

Injury, age related bone defects, as well as pathological conditions are some of the most common impairments related to bone fractures. This usually results in a prolonged healing time, and in some instances, relapse occur due to the treatment not reaching the specific site of action [1]. Current forms of treatment for these defects usually include bone grafts or metallic prosthetic implants. Allografts, xenografts, and autografts are categorized based on their natural tissue source. The most common form of bone implantation therapy is autografts, sampled from the patient’s own body, thereby reducing the risk of tissue rejection. However, this form of therapy is restricted in many cases due to donor site morbidity, long recovery times, as well as substantial tissue damage resulting from surgery [2,3]. Consequently, principles of developing autografts and allograft bone substitutes, using biomaterial of a degradable and biocompatible nature, are increasing owing to the varying biological, structural and physico-mechanical properties that this engineering provides [4]. Research published in 2011 estimates over one million surgical procedures done involving bone defects in the United States (US) per year. This is as a result of trauma, along with non-union healing fractures requiring the implementation of bone grafts. This also affects older patients, thereby incurring greater strain on the healthcare industry, totaling more than 5 billion dollars annually [5,6]. Hence, a significant alternative in the treatment of bone injuries is most certainly required, filling in the gaps of bone grafts, which cannot be undertaken in many instances, due to the limitations of current therapeutic procedures. Bone tissue engineering has shown promising results in this regard. Various scaffold materials are utilized in bone tissue engineering applications, and hydrogels form a major group of these materials [7]. Hydrogels have a hydrophilic nature and therefore have the capacity to absorb several fold their dry weight in water. This allows the cells to adhere and differentiate onto the hydrogels [8]. This form of bone tissue engineering is a multifaceted specialization, involving chemical, biological and material science [9]. The basic element of tissue engineering relies on cells, signals, scaffolds and bioreactors. These cells are three-dimensionally seeded onto a scaffold, cultured in vitro which are supplemented by proper signals, and thereafter implanted as a prosthesis in vivo as outlined in Figure 1 [10]. These cells can be autologous, xenogenic or allogenic, tissue specific stem cells or progenitor cells, which are isolated and cultured in vitro. An extracellular matrix for cell growth, synthetic or natural in composition, is formulated as a scaffold, delivered as a hydrogel. For proliferation and cell differentiation, signals are constructed into the system, enhancing cell adhesion. Bioreactors are also implemented into the system, simulating kinetic responses in the body, which further improve mechanical stimuli and mass transport to emerging tissue [11,12].

To date, injectable hydrogels for application in tissue engineering in the field of orthopedic sciences has produced many benefits over previous application methods. Injectable hydrogel systems are appealing for use in bony defects because they can be formed in situ and have a unique advantage in which difficult sites of injury can be easily targeted with less invasive procedures, forming a composite, irrespective of shape and defect geometry. Hydrogels can be used to fill critical-size bone defects and injuries in non-load bearing sites, even though they do not have the mechanical strength that is needed to provide support for load-bearing functions [13]. Furthermore, this form of delivery can enhance the physico-mechanical properties of injured/pathological bone, delivering a variety of bio-actives, drug molecules, as well as growth factors for therapeutic application [14,15].

Injectable hydrogels represent a remarkable platform for bone tissue engineering due to their state of composition, which has water-based homogeneous properties, and capability of encapsulating, manipulating and transferring their contents to the surrounding tissue, in the least invasive manner [16]. The injected hydrogel, loaded with drug, growth factors and/or cultured cells, reaches its site of action efficiently, and then, further reacting to a stimulus, changes its chemical and physical properties, behaving as a transitional gel-solution system [17]. The properties of the hydrogel promote cellular responses and cell distribution at any site prior to the transitional phase leading to gelation. The substantially hydrated nature allows significant simulation of the extracellular matrix (ECM), due to its similar structural properties. This provides an ideal environment for cellular regeneration and proliferation, thus allowing cells encapsulated in the hydrogel to grow and secrete new ECM for restoration of damaged tissue [18]. Conventional methods for bone tissue regeneration, such as pre-formed hydrogels or scaffolds, face the issue of surgical implantation. The risk of infections and improper adaptation to the injury site using these conventional procedures increases significantly, with the possibility of scaffold failure [19]. Injectable hydrogels are gaining substantial importance in the field of bone tissue engineering by overcoming these challenges, as they can reach the target site with minimum invasiveness, and thus provides a much greater degree of bone repair and regeneration [20].

Thus far, many polymers having specific stimuli responsive behavior and composed of either natural or synthetic materials, designed to effectively deliver their encapsulated contents have been exclusively evaluated for use as thermo-responsive hydrogels, as injectable delivery systems, for applications in bone tissue engineering, due to their biocompatible and biodegradable nature [21]. Natural polymers possess greater biocompatible properties compared to synthetically derived polymers, which usually have to be modified for a biologically structural response [22]. Injectable hydrogel synthesis can be categorized as either physical or chemical gels, in accordance to their gel formulated structure. Physical association crosslinking between polymeric chains or particles in the hydrogel network is regarded as a physical gel system, whereas a chemical gel has covalently bonded polymeric chains in the network system [23]. However, various limitations exist, which include unstable functional groups, the use of cytotoxic reagents, and low coupling efficiency. Thus, an application of more simple, efficient and specific methods of reaction synthesis is required, preserving the biological nature of the bioactive materials, and possessing substantial biocompatible and biodegradable properties. When delivering a hydrogel for bone tissue engineering the bio-kinetics and biodegradable nature of the hydrogel that sustain and improve cellular growth as well as conditions simulated for ECM complementation are of great consideration. To attain these ideal properties, a chemical modification must be undertaken in the structure and density of the crosslinked moieties. Many bio-actives can be attached to the backbone of the polymeric system, thereby improving the function and regulation of cells in the hydrogel system. This paper therefore outlines various techniques of formulating injectable stimuli-responsive hydrogel systems using different natural and synthetic polymers, and their application in bone tissue engineering.

Many approaches exist for the development of an ideal injectable hydrogel for use in bone tissue engineering, capable of increasing healing, adding support, and being biocompatible. The purpose of this review is to provide the reader with a survey of the most current and significant advances in the field of bone tissue engineering, with particular focus on thermo-responsive polymer applications, with various chemical modifications, reporting novel bioengineered delivery systems, currently under evaluation. An area of discussion comprises the various limitations of these delivery systems as well, while the main focus considerably aligns to formulating a system that has exceptional physical, chemical, structural as well as biological advantages, with a unique thermo-responsive nature, enabling the bioengineered system to deliver its loaded content most effectively.

## 2. Approaches to the Synthetic Fabrication of Injectable Hydrogels for Application in Bone Tissue Engineering

Research in the field of chemical crosslinking and physical gelation strategies forms an integral part of the behavioral properties of the hydrogel system for utilization in bone tissue engineering. Physical gelation occurs when two or more polymeric chains form a link due to some physical interaction, such as hydrophobicity, ionic or self-assembly due to environmental stimuli of temperature, pH or polymer precipitation. Chemical crosslinking, on the other hand, provides greater sustained release kinetics, due to the high crosslinking densities of the polymeric network, providing complimentary mechanical properties for scaffold-based delivery. A drawback of this, however, is the nature of the crosslinking agents used in the reactions, which often possess toxic chemical profiles, affecting bioactive materials and cells which are incorporated into the hydrogel. Physical gelation may avoid the use of crosslinking agents, though demonstrating less physical effective properties. The various mechanisms of solidification of injectable hydrogels for application in bone tissue engineering in response to the diverse environmental stimuli will be discussed in this section.

### 2.1. Thermo-Induced Gelation: Classical Poly(NIPAAm)

Thermally-induced gelation systems (TGS) are uniquely composed of an interpenetrating network of polymeric chains that are able to undergo phase transitions to the gel-sol state, due to the manipulation of temperature. The polymeric chains are amphiphilic in nature, being based of an interaction between hydrophobic and intermolecular forces, on which the molecular weights of these segments are responsible. In both chemical and physical polymeric systems, drugs are loaded at room temperature. Once the formulation is injected, the gel shrinks, entrapping the loaded contents, modulating their release. One of the proposed mechanisms for thermal transition include the shrinkage of the outer shell, forming a barrier, reducing the pore size of the gel, thereby reducing the diffusion rate of the water molecules. One of the oldest polymers for biomedical administration is Poly(*N*-isopropylacrylamide) (PNIPAAm). This is due to its lower critical solution temperature (LCST) being very close to body temperature. Crosslinking of this polymer causes a coil to globule transition, thereby decreasing the gel volume, releasing the entrapped drug, following a linear controlled release rate [24]. It was also reported that hydrophobic drugs strengthen the structure of the hydrogel system, and by varying the crosslinker concentration, the release rate of the drug from PNIPAAm gels could be altered [25]. This polymer also demonstrated a considerable change in LCST due to the salt concentration in the polymeric network, lowering the LCST of the polymer, due to the Hofmeister effect on water molecules [26].

**Figure 2:**
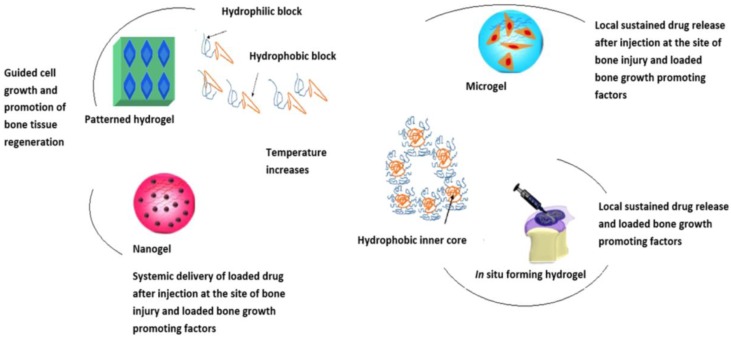
Physical gelation system derived from increased temperature due to interaction of hydrophilic and hydrophobic blocks for promoting an amphiphilic polymer.

Research undertaken demonstrated that co-networks of PNIPAAm and hydroxyethyl methacrylate (HEMA) resulted in an LCST of 34 °C, resulting in favourable in vivo kinetics for drug release [27]. A similar study was undertaken by crosslinking zinc tetraphenylporphyrin (Zn-TPP) into PNIPAAm-co-PHEMA. Results revealed that above the LCST, Zn-TPP release was significantly decreased, thereby controlling the kinetics due to polymer concentration and manipulation of the LCST [28]. Studies have been undertaken by crosslinking PNIPAAm with polyamino acids, resulting in thermo-responsive hydrogels [29]. Further research in this field reported the synthesis of elastin based thermo-responsive polymers, existing in the solvated state below their transition temperature, and above its LCST of 30 °C, the polymeric chains change conformation to form nanoparticles, entrapping molecules to deliver the actives, such as a loaded-drugs, proteins or cell cultured systems. They are also nontoxic and do not produce an immunogenic response once delivered, forming an amphiphilic copolymeric chain [30,31]. Figure 2 further demonstrates the gelation mechanism brought about from the change in temperature, creating an amphiphilic copolymeric chain loaded with cells and drug molecules as desired for specific injectable technologies.

Bessa and co-workers also reported that morphogenetic proteins could be delivered at a controlled rate for fourteen days, consequently enhancing bone formation [30]. Another study undertaken by grafting PNIPAAm and PVCL-HEMA on a dextran chain, produced a thermos-responsive biodegradable hydrogel with sustained drug release for numerous days, with no immunological responses [32]. Hydrogels incorporating PEGMA and iron oxide displayed a positive thermos-responsive drug delivery application by deswelling once the temperature was increased. They also displayed magnetic properties by heating upon application of an alternating magnetic field, leading to deswelling of the hydrogel [33]. Similarly, another study reported hydrogels possessing super-paramagnetic properties, incorporating magnetite nanoparticles through polymerization, demonstrating tunable super-paramagnetic properties proportional to the concentration of magnetite by increasing the stimuli of temperature [34]. Research undertaken by Ma et al., demonstrated a hydrogel of PNIPAAm (constituting 80% and greater of the polymer concentration), PHEMA, incorporating a monomer of lactic acid, which then produced LCSTs in the range of 10–20 °C, possessing significant tensile strength, while degrading over several months when used for bone tissue engineering application [35].

Among other polymers, methylcellulose has been studied for decades, demonstrating its good thermo-responsive properties. A study demonstrated that conjugation of methylcellulose with the protein laminin produced a sustainable hydrogel for neural defective tissue, increasing cell adhesion by the addition of a protein conjugate [36]. A series of PEGylated polymers were also studied for tissue engineering. Further research evaluation demonstrated that by implementing PEG-alt-thiol conjugates, this biodegradable polymeric system showed in vivo biocompatible cell matrices [37]. Conjugation of pluronics with peptides increases cell adhesion and growth responses in scaffold formulations [38]. Kan et al. delivered a zero order drug delivery thermo-responsive polymer PEG-b-PLGA-b-PEG, forming an oil in water emulsion. The hydrophilic phase displayed a gel at 20–30 °C, above the polymer LCST, preventing the drug from erupting, and encapsulating the oil droplets for controlled drug release [39].

The most significant factor in all of the above polymeric systems is the level of solubility that is attained above the LCST, due to the entanglement and collapse of the polymeric network chains. Factors such as polymer concentration, chemical structure and molecular weight affect the gelation process. Other examples of polymers implemented as thermo-sensitive hydrogels include poly(propylene oxide), poly(ethylene oxide), poloxamers or pluronics, chitosan, gelatin as well as cellulose derivatives which have been identified to a great extent in the field of drug delivery and bone tissue engineering.

Research undertaken at the University of California, Berkeley, reported osteogenic differentiation via soft bioinspired hydrogels [40]. Many physical and biochemical factors guide the osteogenic differentiation of human mesenchymal stem cells. Modulus (rigidity) of the ECM is one factor which has gained much attention as a physical osteo-inductive signal that can contribute to endochondral ossification of a cartilaginous skeletal template. To understand the function of the matrix interactions in this process, osteogenic differentiation of human mesenchymal stem cells cultured on low moduli (102 Pa) poly(NIPAAm) based semi-interpenetrating networks, modified with the integrin engaging peptide bsp-RGD (105 µM), was analyzed. It was concluded that within the low moduli poly(NIPAAm) substrate, a very high affinity adhesive ligand serves as a substitute for a rigid matrix to promote osteogenic differentiation. In addition to this research, various other studies using this polymer evaluated its application in bone tissue engineering, with similar positive results. Thus it can be deduced that poly(NIPAAm) demonstrates promising results in the field of bone tissue engineering.

### 2.2. Ionic-Mediated Gelation: The Stimulation of Alginate

This system is mediated by the formation of ionic chain connections with di- or trivalent cations of the polymeric network. Alginate is the most studied polymer, with its ability to crosslink with calcium and zinc cations at different positions of the alginate chain, due to its polysaccharide structure, composed of homopolymeric blocks of 1,4-linked β-d-mannuronic acid and α-l-guluronic acid [41]. The polyguluronic acid block makes this polymer more selective for binding to calcium ions. Zinc cations are less selective for crosslinking, resulting in greater zinc alginate crosslinked hydrogel systems [42]. The rate of crosslinking has been reported to be inversely proportional to the concentration of alginate used and the rate of crosslinking influenced by the concentration of multivalent cations and polyguluronic acid segments present [43].

A remarkable study undertaken by Roman et al., evaluated a core–shell fibrous collagen-alginate hydrogel cell delivery system for bone tissue engineering [44]. In this study, a novel stem cell delivery system constituted of alginate and collagen as the core and shell was designed. Mesenchymal stem cells were loaded into the collagen solution. This was then placed immediately into a fibrous structure while concurrently sheathing with alginate by use of a newly designed core-shell nozzle. The continuous fibrous structure of the inner cell-collagen part was successfully maintained, as the alginate encapsulation was attained by the crosslinking within an adjusted calcium-containing solution. A continuous fiber with a diameter of ~700–1000 μm for the core and 200–500 μm for the shell area was displayed by the constructed hydrogel carriers. This was greatly dependent on the alginate concentration (2%–5%) and the injection rate (20–80 mL/h). The mesenchymal stem cells which were encapsulated within the collagen exhibited exceptional viability. It also displayed remarkable cellular proliferation for up to 21 days with levels comparable to pure collagen gel matrix which was used as a control. The cells which were allowed to differentiate under osteogenic conditions displayed favorable levels of bone-related genes, comprising of osteocalcin, osteopontin and bone sialoprotein. Bone healing was considerably enhanced when the core-shell fibrous carriers were implanted in a rat calvarium defect, more so after an osteogenic induction of the mesenchymal stem cells before implantation. Based on the results of this experiment, the newly fabricated core–shell collagen-alginate fibrous carrier is regarded as favorable to permit the encapsulation of tissue cells and their delivery into damaged tissues, including bone with defect-tunability for bone tissue engineering.

### 2.3. Free Radical Polymerized Gelation: The Essential Vinyl Monomer

**Figure 3:**
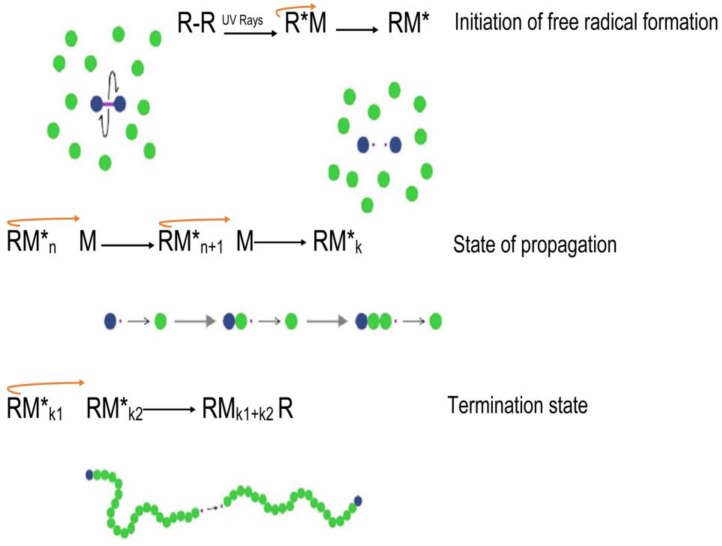
Free radical polymerization steps involved in copolymerization techniques.

Free radical polymerization techniques are commonly generated by radical generating initiators such as gamma radiation, ultraviolet light as well as redox initiators. This technique involves a radical-generating initiator system and a radical-labile oligomer or monomer [45]. This fundamental process proceeds only by the presence of free-radical initiation. Vinyl groups commonly generate free radicals upon physiological stimuli, containing radical-labile moieties, which further undergo propagation steps in the polymerization network formation. After completing the initiation phase, radical initiators homolytically cleave the radical-labile moieties, inducing the propagation phase, as seen in Figure 3. The final step takes place as a termination step by which two radicals in the propagation polymer bond covalently, thereby completing the crosslinking process [45]. The efficiency of the hydrogel can be evaluated by its mechanical properties, crosslinking density as well as its biodegradable profiles, which are dependent on the radical-labile moieties, nature of the solvents used and the concentration of the initiator. Redox initiated systems are preferred in areas of limited light rays where crosslinking of the polymeric networks are homogenous, and photo-initiated systems are preferred where spatial and temporal control are essential to develop structured patterned complexes [46].

These free radical polymerization reactions are essential methods in synthesizing various thermo-responsive copolymers. Using a crosslinker and an initiator in the reactions, successful copolymerization can occur under favorable conditions. Application of these reactions for polymeric scaffold design using 3D bio-plotting is currently a huge focus for incorporation of cell cultured systems, delivering a host of growth factors and drugs for the treatment of bone related injuries. These thermo-responsive scaffolds swell at body temperature, with great biocompatibility properties, introducing tissue cells and growth factors at the site of injectable delivery. At body temperatures, these free radical copolymerized polymers begin to change from the solution to gel state, further providing mechanical support and significant matrix resilience at the site of bone injury [45,46].

A recent study described a thermoplastic starch/ethylene vinyl alcohol/forsterite nanocomposite as a candidate material for bone tissue engineering [47]. The aim of the study was to synthesize a nanocomposite biomaterial which was composed of a blend of thermoplastic starch and ethylene vinyl alcohol as the polymer matrix. Nano-structured forsterite was used as the ceramic reinforcing phase for bone tissue engineering. It was found that blending of thermoplastic starch and ethylene vinyl alcohol modified the degradation rate and water resorption of thermoplastic starch. The nanoforsterite improved the mechanical and biological traits and decreased the weight loss of the thermoplastic starch and ethylene vinyl alcohol blend in simulated body fluid. Furthermore, this modified the pH in the methylthiazolyl tetrazolium (MTT) assay and prompted cell proliferation. A favorable interaction between cells and the biomaterial was demonstrated by cell adhesion assays. It was concluded that the proposed nanocomposite displays pertinent biocompatibility and mechanical properties and can be used in bone tissue engineering.

### 2.4. Self-Assembled Gelation: The Independent Amphiphiles

Many amphiphilic polymers display gelation properties due to their complexation as a result of desolvation, collapse and intermolecular association of nonpolar segments of the monomers. Further stability is displayed when these amphiphiles are charged, possessing electrostatic and van der Waals forces [48]. Various peptide bio-actives display amphiphilic properties in injectable tissue engineering, with the hydrogel displaying low viscous properties at lower temperature, and changing its conformation to a gel form once injected in vivo [49]. Hydrophobic polymers also display self-assembly via phase segregation, in which water soluble solvents have been injected with these biodegradable polymers [50]. A precipitation reaction occurs when water diffuses into the polymer matrix and solvent diffuses into the tissue space, resulting in a hydrophobic polymeric complex at the site of injury into the injected cavity. Factors affecting the rate of precipitation include the molecular weight of the polymer, concentration of polymer in solvent, addition of a surfactant and the type of solvent used [51]. Many biologicals have been encapsulated and delivered, such as bone morphogenetic protein-2 (BMP-2) and fibroblast growth factor (bFGF), formulating a suspension of these bio-actives in aqueous solution of amphiphilic peptide, forming a self-assembly hydrogel system. Some examples of solvents used in this approach are *N*-methyl-2-pyrrolidone, propylene glycol, dimethyl sulfoxide, acetone, 2-pyrrolidone and tetrahydrofuran [52]. This system is indeed useful in incorporating biological actives and peptide based therapeutics, increasing its stability and mechanical structural properties by using a good selection of amphiphilic polymers with the correct type of solvents.

### 2.5. Chemically Crosslinked Gelation: The Sentimental Alkynes, Azides and Amines

Polymers containing high affinity for each other and displaying significant solubility in the injectable medium can be modified to form a covalently linked network at the site of delivery [53]. Most frequently reported molecules having this relationship include, alkyne to azides, *N*-hydroxy-succinimide (–NHS) to amines (–NH_2_) and an α,β-unsaturated carbonyl compound in the presence of a 1,4-addition carbon nucleophile. The degree of network formation, stability, and kinetics of the system are proportional to the affinity of molecules. Much research has been conducted on amine-based polymers in the field of bone tissue engineering. Researchers investigated the in vitro and in vivo osteogenic activity of the novel vancomycin-loaded bone-like hydroxyapatite/poly(amino acid) scaffold [54]. Bone-like hydroxyapatite/poly(amino acid) scaffolds have been shown to display some osteogenic and osteo-conductive properties. It also exhibits controllable biodegradability, and nontoxicity. A homogenous method which utilized a diffusion control system was used to successfully produce a vancomycin-loaded bone-like hydroxyapatite/poly(amino acid). In vitro tests included incubating MG63 cells with the vancomycin-loaded scaffold. This was done to observe its effect on the activation of osteoblast cells. The in vivo tests were conducted on a rabbit model. The scaffolds were implanted into the rabbit model which possessed chronic osteomyelitis. The MG63 cells displayed good proliferative activity and increased calcium and phosphatase synthesis. In vivo test results showed an increase in bone growth in infectious bone defects as compared to the control group, regardless of the type of *Staphylococcus aureus* present. It was thus concluded that due to its ability to deliver antibiotics and promote bone regeneration, vancomycin-loaded bone-like hydroxyapatite/poly(amino acid) has great potential for the repair of infectious bone defects [54].

Polymers can also be crosslinked by enzymatic reactions, by which phenol groups undergo self-crosslinking in the presence of hydrogen peroxide (H_2_O_2_) and horseradish peroxidase (HRP). When proteins react with H_2_O_2_ and peroxidase, oxidation of the phenol groups of tyrosine occurs, forming a crosslinked protein conjugate of di- and tertyrosine [55]. Polymers such as chitosan, dextran, hyaluronic acid and gelatin can introduce a phenol group on its structure by reacting their carboxylic or amine groups in the presence of 3,4-hydroxyphenylpropionic acid (HPA) or tyramine [56]. Poly(lysine-co-phenylalanine) and transglutaminase (TG) mediated glutaminamide-functionalized poly(ethylene glycol) (PEG) have also been reported to display chemical crosslinking properties in place of HRP, employing calcium ions as cofactors [57]. The rate of crosslinking of the system can be attributed to the phenol functionality and the concentration of HRP and H_2_O_2_, however an excess of H_2_O_2_ can deactivate the crosslinking reaction by oxidizing HRP [58]. This network formation therefore has great potential in tissue engineered hydrogel systems when incorporating protein derived bio-actives, forming stable networks of chemically crosslinked polymers.

## 3. Polymers Employed for the Synthesis of Stimuli-Responsive Hydrogel Systems

Various biocompatibility techniques for the delivery of natural and synthetic polymers can be explored by their structural activity relationship, forming stimuli responsive hydrogel systems. Polymers such as hyaluronic acid, chitosan, matrigel, collagen and agarose are natural polymers that can be modified for specialised application. Synthetic polymers such as poly(ethylene glycol) (PEG) and PNIPAAm are examples of modified release block polymers which can be crosslinked to naturally derived polymers to serve as hydrogel responsive carrier systems for biologicals and drug delivery.

### 3.1. Natural Polymers

#### 3.1.1. Hyaluronic Acid

This glycosaminoglycan found in the extracellular matrix is often used as a key component in hydrogel systems used as a delivery carrier for drugs and protein molecules. This is due to its biodegradable and biocompatible nature, forming a gel with substantial water content [58]. Hyaluronic acid (HA) can be enzymatically degraded, and therefore does not result in an immune responsive cascade, thus being effectively delivered as an injectable delivery system. HA is not thermo-responsive in nature, however, it is most often conjugated with thermo-responsive polymers to provide a modified hydrogel, being biodegradable, biocompatible and thermo-responsive [59]. Many publications have demonstrated the conjugation of PNIPAAm with HA, displaying admirable properties as a thermo-sensitive hydrogel [60]. The thermal transition temperature of the conjugated HA-PNIPAAm was independent of the molecular mass of HA, PNIPAAm grafting ratio and its chain length, maintaining the poor reactivity in cell adhesion, being highly applicable in tissue grafting technology [61]. To improve the cell adhesive properties of HA, researchers have conjugated HA with stimuli responsive polymers such as PNIPAAm, pluronic and gelatin [62]. Pluronic has significant properties in response to temperature, which can be chemically or physically modified to HA. Its copolymeric structure has chains of poly(ethylene oxide)-β-poly(propylene oxide)-β-poly(ethylene oxide) (PEO-PPO-PEO). HA-Pluronic copolymeric hydrogels displayed admirable drug release properties, delivering chemotherapeutic drugs following a Fickian diffusive mechanism [63]. It can therefore be affirmed that the properties of HA offer substantial advantages in the delivery of bioactive formulations, further conjugated to stimuli responsive polymers to yield biocompatible and biodegradable injectable technologies.

The development of an injectable calcium phosphate/hyaluronic acid microparticles system for platelet lysate sustained delivery aiming at bone regeneration was designed and evaluated [64]. The incorporation of calcium phosphate (CaP) cement in conjunction with HA microparticles loaded with platelet lysate (PL) to enhance the degradability and biological performance of the proposed cements was investigated. Cement formulations were developed, which consisted of increasing weight ratios of either HA microparticles or microparticles loaded with PL (10 and 20 wt %). Formulations with cements directly incorporating PL were also fabricated. Homogeneous particles distribution as well as increased interconnectivity between the HA microparticles were exhibited by morphological analysis. A sustained release of 8 days was observed with the cements incorporating PL proteins. This sustained release of PL modulates the expression markers in seeded human adipose tissue- derived stem cells. This therefore suggests the stimulatory role of this combination system toward osteogenic commitment as well as bone regeneration and repair applications.

#### 3.1.2. Chitosan-Based Hydrogels

The polysaccharide chitin, derived from the exoskeleton of crustaceans and insects, is one of the most abundant naturally available polymers. Chitosan is derived from chitin by deactylation reactions of *N*-acetyl-d-glucosamine [65]. Chitosan is not a thermo-sensitive polymer, however the addition of glycerophosphate (GP) in solution of chitosan, provides a thermo-sensitive hydrogel, by which the pH of the solution must be less than 6 for the solubility of the chitosan polymer. GP reacts with chitosan to form hydrogen bonds when the temperature is increased, thereby leading to a gel formation. The different drug loading and release properties of chitosan were studied with various gelation times and drug release kinetics for various chitosan conjugated systems. Chitosan-GP copolymer was studied in vivo in a rodent model, delivering bone morphogenetic protein (BMP) for cartilage repair. The bioactive loaded hydrogel system was injected subcutaneously and was observed to show a gelling time of 10 min. This application demonstrated a low degree of success for delivering the loaded bioactive due to the extensive time of gelation [66]. To enhance the duration of gelation, an approach of obtaining chitosan chloride with GP was investigated, displaying greater solubility than chitosan, with a gelation time of 1 min. This system was investigated to deliver insulin, retaining the protein efficiency, displaying success as an injectable hydrogel delivery system for diabetes [67]. This however has a disadvantage of the rapid release rate of drugs and protein molecules. The study showed that within a few hours, all of the drug was released with no sustained kinetic profiles, proving its inefficiency to deliver long term controlled formulations. Another strategy of interest was to encapsulate a low molecular weight molecule using liposomes in the chitosan-GP hydrogel system. The drug carboxyfluorescein was used to determine the release behaviour of the hydrogel, displaying a sustained kinetic profile of 2 weeks [68]. It was noted that the liposome addition did not change the gel transition temperature, however, the size of the liposomes affected the release rate of the drug. Another manipulation strategy to delay the release rates of the loaded bioactive was investigated, by which silicon nanoparticles (SNPs) were used to encapsulate ovalbumin from a chicken, suspended in a chitosan-GP hydrogel system. Results obtained demonstrated a 40% release rate at day 14, in comparison to a 100% release without encapsulation of SNP. It can be explained that the SNP hindered the release, due to possible temperature and nanoparticle interaction with the protein, preventing substantial release from taking place at once [69]. A disadvantage of the above mentioned study was further reported, by which toxicology of chitosan-GP hydrogels were described. Foreign body immune modulated responses were detected when the hydrogel formulation was administered. Different ratios of chitosan-GP concentrations, as well as adding anti-inflammatory agents did not hinder the immune response, hence allowing detection of foreign materials in vivo [70]. It can therefore be deduced that chitosan has significant potential for delivery of bioactive molecules by conjugating to various stimuli responsive polymers, or using various carrier strategies to effectively manipulate the release kinetics, providing a variety of mechanisms for successful injectable delivery.

The modification of chitosan side chains can provide a diversity of derivatives for bone tissue engineering applications [71]. Chitosan was modified into *N*,*N*,*N*-trimethylchitosan (TMC) by reaction with methyl iodide and in another example, *N*-(2-hydroxyl) propyl-3-trimethylammonium chitosan chloride (HTCC) was prepared by dissolving chitosan in NaOH/CHPTAC aqueous solution by a pressure-equalizing dropping funnel [72]. Other modifications of chitosan include *N*-(2-hydroxyl) propyl-3-triethylammonium chitosan chloride (HTEC) and *N*-(2-hydroxyphenyl)-*N*,*N*-dimethylchitosan (NHPDCS) [73]. Disrupting the negatively charged outer membrane of microbes is how chitosan exerts its antimicrobial activity. This is one of the many important characteristics that is needed in bone tissue engineering, and is currently still being evaluated in various bone tissue engineered delivery hydrogel systems, due to substantial biocompatible, tissue regenerative properties.

#### 3.1.3. Cellulose-Derived Hydrogels

Cellulose is composed of repeating units of β-(1,4)-d-glucose, an elementary component of plant cell walls, being the most abundant natural polymer available [74]. This polymer does not possess thermo-sensitive properties, however modification of hydrophobic groups on cellulose makes the polymer thermo-sensitive. It is widely used in skin tissue and wound healing engineering. The incorporation of alkyl groups on cellulose provides significant thermo-sensitivity, proportional to the gelation rate of the hydrogel [75]. Blending was also investigated with cellulose using other natural and synthetic polymers to provide thermo-sensitivity. Alginate salts and chitosan displayed thermo-responsive properties when blended to cellulose, however, chitosan-carboxymethyl cellulose (CMC) hydrogel displayed both thermo-sensitive and pH responsive behavior [76]. An alginate/ hydroxypropyl methyl cellulose (HPMC) copolymeric hydrogel was also investigated, displaying a controlled release rate of 400 h, using a prototype heparin drug [77]. Similar studies were carried out using synthetic polymers of CMC blended with poly(*N*-vinyl pyrrolidone) (PVP), which also displayed pH and thermo-sensitive properties. The gelation was found to occur between 24 and 29 °C, although the release of the loaded bovine serum albumin from the hydrogel was greater as the pH transitioned from acidic to basic [78]. Cellulose certainly has great potential for injectable delivery of bio-actives, since its amphiphilic properties, through conjugation with other polymers, result in highly responsive characteristics, allowing controlled release rates and maximum delivery of its loaded bioactive molecules.

A study conducted by Deniz and co-workers, looked at crosslinked pullulan/cellulose acetate fibrous scaffolds for bone tissue engineering [79]. Pullulan (P) and cellulose acetate (CA) were electrospun at different P/CA ratios (P80/CA20, P50/CA50, and P20/CA80) to fabricate a 3D fibrous network. To enhance the mechanical properties, the scaffolds were thereafter crosslinked with trisodium trimetaphosphate (STMP). This also delayed rapid weight loss. Groups that were crosslinked with P/STMP 2/1 for 10 min displayed the lowest weight loss. Fiber morphologies were more uniform with the P50/CA50 group, without phase separation. This group was also crosslinked more effectively than the other groups. Crosslinked P50/CA50 scaffolds, among all groups, had more uniform pores. This group was thus used for bio-activity and cell culture studies. After incubation, apatite-like structures were noticed on the fibers. Human osteogenic sarcoma cell line seeded onto the crosslinked adhered and proliferated P50/CA50 scaffolds. ALP activity of the cells were used to measure the functionality of the cells. The results demonstrated their osteoblastic differentiation. It was concluded that the crosslinked P50/CA50 scaffolds were the best candidate cell carrier for bone tissue engineering in this study, with similar correlations reported by many researchers to date.

#### 3.1.4. Other Natural Polymers

Other naturally available polymers are gelatin, collagen, agarose and matrigel, which also possess thermo-sensitive properties. Gelatin, collagen and agarose, however, possess negative thermo-sensitive properties, forming a gel at lower temperature and having liquid state properties at higher temperature conditions. Matrigel on the other hand, has positive thermo-sensitivity, solidifying at higher temperatures. This polymer is a mixture of components of basement membrane compounds, derived from chondrosarcoma, which has also been shown to display negative carcinogenic effects in vivo, due to its tumor-based origin [80,81]. Matrigel is a trademarked product of the Becton Dickinson company, which was shown to have significant cytocompatibility, allowing cells such as endothelial and chondrocytes to turn into a functional phenotype on the matrigel. It has been used as a cellular simulated environment for evaluating immune responses to drugs and other bioactive molecules. Anticancer loaded drugs in matrigel have been shown to inhibit the progression of tumours, thereby displaying a liquid characteristic at 4 °C, forming a gel-like substance at body temperature that swells to release its loaded bioactive molecules [82,83]. It can be considered that these natural polymers have characteristics that are essential in simulating biocompatible matrices, however their limitations allow for using synthetic polymers in combination to deliver refined, highly specific stimuli responsive bioactive loaded copolymeric system.

The main component of connective tissue in mammals is collagen. Collagen type 1 exists in the form of elongated fibrils in bone [84]. It is the abundant in nature and has been considered for various bone tissue engineering applications as it has low antigenicity and excellent biocompatibility. Another reason why collagen is considered in many bone tissue engineering applications is that it has good crosslinking abilities, thus its mechanical and degradation properties can be modified.

Enzymatically crosslinked carboxymethylchitosan/gelatin/nanohydroxyapatite injectable gels for in situ bone tissue engineering applications were studied by Mishra et al. [85]. The results of this study revealed that at physiological temperature, a combination of *p*-cresol (2 mM) and tyrosinase (60 Units), as crosslinkers, yield rigid gels when applied to carboxymethylchitosan/gelatin within 35 min in the presence of nanohydroxyapatite. An increase in carboxymethylchitosan concentration in the gel was observed by FTIR and rheological analysis. This leads to increased strength as well as a greater degree of crosslinking. SEM analysis revealed that the pore sizes of the injectable gels increased with higher concentrations of gelatin. In vivo application if the injectable gels in mice showed that the degree of crosslinking and carboxymethylchitosan concentration determined the stability of the in situ formed gels. The study concluded that the carboxymethyl–chitosan/gelatin/nanohydroxyapatite injectable gels could be used for bone cell delivery as well as in the treatment of irregular small bone defects with very little clinical invasion.

A study conducted by Puértolas et al., researched compression behavior of biphasic calcium phosphate and biphasic calcium phosphate–agarose scaffolds for bone regeneration [86]. In order to study the potential applications of biphasic calcium phosphate (BCP) bioceramics and BCP-agarose systems for bone tissue engineering, static and dynamic compression tests have been assessed. A brittle behavior was observed for the dense and designed porous design scaffolds in the BCP systems. Agarose displayed toughness, ductility as well as a rubbery consistency up to 60% in bioceramic BCP-agarose systems. This resulted in maximum strength, without losing its initial cylindrical structure, of 10–50 MPa. A greater mechanical resistance was displayed by dried and rehydrated BCP-agarose systems, which is substantially higher than standard hydrogel systems. The BCP-agarose system is very promising for bone tissue engineering because it has excellent mechanical properties, as well as exceptional biocompatibility, biodegradability and affinity for proteins and cells.

### 3.2. Synthetic Polymers

Due to the lack in versatility and responsiveness in natural polymers as thermo-sensitive hydrogel carriers, synthetically derived polymers have been used as the basis for modification and conjugation with natural polymers, allowing chemical alteration in the design and responsiveness of the hydrogel system. Due to the complexity of specific carrier systems, natural polymers may not have the capacity to deliver the loaded bioactive efficiently. Synthetic thermo-sensitive polymers can be designed with flexibility for reactivity, release kinetics, gelation temperature, as well as biodegradation properties. This section will discuss the various synthetic based derivatives as thermo-sensitive hydrogel systems.

#### 3.2.1. Polyacrylamide-Derived Thermo-Responsive Hydrogels

Acrylamide-based polymers have been studied for decades, being applicable to deliver thermo-responsive bioactive molecules, manipulating the lower critical solution temperature (LCST), forming a homogenous injectable delivery system [87,88]. *N*-substituted polyacrylamides such as poly(*N*,*N*-dimethylacrylamide), poly(*N*-(2-hydroxypropyl) methacrylamide lactate, poly(*N*-vinyl caprolactam) and PNIPAAm have significant potential as injectable hydrogel delivery systems, where molecules exist in the hydrated form due to their *N*-substituted hydrophobic functional groups [89,90]. When the temperature is above LCST, interaction between *N*-substituted hydrophobic groups’ increase above hydration energy, leading to crosslinking of the polymeric chains, forming a hydrogel dispersed network of particles. Swelling of the hydrogel system occur bellow the volume phase transition temperature (VPTT) and breaks down above VPTT [91]. The most widely used polyacrylamide is PNIPAAm, with significant properties in response to the physiological environment, forming a hydrogel at 32 °C, being able to deliver a variety of bioactive molecules as an injectable delivery system [92].

This section will explore the various research techniques undertaken for advancing the reactivity, LCST, swelling properties and biodegradable nature of PNIPAAm and its crosslinked copolymers. One of the drawbacks of using pure PNIPAAm hydrogels, is the instability in hydrophilic environments, which makes them dissolve rapidly, thereby releasing their loaded bioactive molecules at a faster rate than desired. Polymers such as *N*,*N*-methylenebisacrylamide (MBAAm) and 2-hydroxyethyl methacrylate (HEMA), which have two double bonds, are usually crosslinked to provide a greater stability ratio for a controlled release delivery system [93,94].

Interpenetrating network (IPN) formation is another technique for prolonging the release of PNIPAAm based polymers. Silk fibroin/PNIPAAm and NIPAAm/MBAAm IPN polymerization solution displayed greater stability than non-IPN formulations [95]. Another technique was copolymerization of *N*-isopropylacrylamide (NIPAAm) with monomers such as oligolactide-(2-hydroxymethyl methacrylate) (oligoLA-HEMA), which successfully delivered insulin to the retina, displaying high encapsulation and sustained release rates over a 7-day period [96]. A significant limitation to this versatile polymer is the inability to degrade under physiological conditions, above 32 °C. Cho et al. used strategies to copolymerize biodegradable polymers to PNIPAAm by altering the LCST of the hydrogel using PLA and l-lysine [97]. A similar approach was undertaken by Zhao et al. incorporating poly(NIPAAm-co-HEMA) and biodegradable poly(l-glutamic acid), which possessed biodegradable properties as well as pH and thermo-sensitive characteristics [98]. In conjunction with the success of deriving a biodegradable system, the limitation of toxic degradation products of low-molecular mass PNIPAAm displayed cytotoxicity in reproductive cells [99]. In an attempt to combat these toxicological profiles, research was conducted using PNIPAAm biodegradable copolymers, having various LCSTs before and after degradation due to its biodegradable hydrophobic side chains, which have gelation temperatures below 37 °C before degrading, forming hydrogels in the body. After degradation of the side chains, the gelation temperature increased above 37 °C, dissolving the degraded products in the body fluid, thereby being removed from the body, and remaining in higher molecular mass due to unchanging of the backbone of the conjugated copolymer [100]. It is thus believed that these copolymeric hydrogels have great potential in injectable delivery systems, due to their ability to significantly manipulate the gelation temperatures of the copolymer and its by-products derived under physiological conditions.

#### 3.2.2. Poly(Oligo (Ethylene Glycol) Methacrylate)-Derived Thermo-Sensitive Hydrogels

Poly(oligo (ethylene glycol) methacrylate) (POEGMA)-derived hydrogels have thermo-sensitive and biocompatible properties. This hydrogel system has been reported to deliver a variety of chemotherapeutic drugs [101]. This system can be modified for drug release and variation in thermal response. It was reported that protein loaded in this polymer showed nonspecific protein adsorption, being applicable for non-protein loaded delivery systems. Oligo(ethylene glycol) methacrylate (OEGMA) and 2-(2-methoxyethoxy)ethyl methacrylate (MEO_2_MA) copolymerization demonstrated gelation temperatures between 26 and 90 °C, having a vast degree of application in biomedical and chemically-derived injectable delivery systems. The alteration in the concentration of OEGMA contributes to the LCST of the hydrogel [102,103]. This system is however not applicable to injectable cell delivery, but highly beneficial for drug delivery of non-protein systems.

#### 3.2.3. Polyphosphazene-Derived Thermo-Sensitive Hydrogels

Polyphosphazenes are desirable polymers for thermo-sensitive delivery systems, having immense potential for chemical modification due to their alternating single and double bonds of nitrogen and phosphorus functionalities. This makes them targets for conjugation with other polymers, contributing to their biocompatible nature [104,105]. The side chains can be modified to obtain poly(organophosphazene)s with α-amino-ω-methoxypoly(ethyleneglycol) which is hydrophilic in nature and hydrophobic l-isoleucine ethyl ester (IleOEt), which can be altered to have thermo-sensitive and pH responsive properties with various gelation temperatures [106]. The degradation products of polyphosphazenes, which results in alcohol, ammonia and phosphate by-products that are non-toxic, therefore makes them readily biocompatible in vivo, which is especially seen in chemotherapeutics [107]. It can therefore be inferred that this hydrogel system has good loading, release and gelation properties, providing a versatile system for the delivery of many bio-actives and drug delivery systems [108].

#### 3.2.4. Pluronic-Derived Thermo-Sensitive Hydrogels

The thermo-sensitive copolymer PEO-PPO-PEO, commonly referred to as pluronic, has various useful properties such as adjustable block lengths, a liquid state at lower temperatures due to the interaction with PEO and formation of a gel at higher temperatures due to hydrophobic interactions [109]. Pluronic hydrogels are unstable in physiologically simulated environments, having a rapid dissolution, as a result, making the hydrogel limited for application in control release systems [110]. To decrease the release from the delivery system, chemical crosslinking can be undertaken, incorporating thiol and acrylate groups on either side of the chemical chain, increasing the drug retention time and the stability of the hydrogel [111]. It was also found that by crosslinking these groups to pluronic, the loading capacity of the drug was substantially increased [112]. Chemical modification can easily be achieved due to the terminal hydroxyl functionalities on the chain. Studies undertaken on chemotherapeutic drugs displayed increased retention times, and greater sensitivity to tumours, by conjugating with linoleic acid, once injected at the site of the tumor [113]. Studies carried out on antithrombotic drugs showed similar results, having a high encapsulation and a sustained zero order delivery rate, with higher bioactivity [114]. The main area of concern for this polymer is its biodegradability, whereby its polyether chains cannot degrade under physiological conditions, limiting their application in many injectable hydrogel delivery systems. Their application in protein therapeutics of drug delivery has been noted [115], however, there is much to be considered when the polymer is not biodegradable for in vivo application, when applied to injectable drug delivery.

Tissue-engineered bone formation using periosteal-derived cells and polydioxanone/pluronic F127 scaffold (PDO/Pluronic F127), with pre-seeded adipose tissue-derived CD146 positive endothelial-like cells were studied by Jin-Ho et al., from the Department of Advanced Materials, Hannam University, South Korea [116]. CD146 positive adipose tissue-derived cells were sorted to purify more endothelial cells in characterization, considering the hematopoietic and mesenchymal phenotypes of adipose tissue-derived cells cultured in EBM-2 medium. These cells were indicated as adipose tissue-derived CD146 positive endothelial-like cells. A great source of osteogenic cells for tissue engineered bone formation is periosteum. In a PDO/Pluronic F127 scaffold, Periosteal-derived cells were established to possess great osteogenic capacity. This could provide a fitting environment for osteoblastic differentiation of these cells. These cells could be investigated in EBM-2 with osteogenic induction factors through the study of capillary-like tube formation on matrigel and the cellular proliferation of adipose tissue-derived CD146 positive endothelial-like cells cultured in various media conditions. It was also observed that in the early period of culture, the osteogenic activity of periosteal-derived cells is satisfactory in EBM-2 with osteogenic induction factors. In vivo studies in a pig model revealed that tissue-engineered bone formation can be utilized to restore the bony defects of the maxillofacial region using periosteal-derived cells and PDO/Pluronic F127 scaffold with pre-seeded adipose tissue-derived CD146 positive endothelial-like cells.

#### 3.2.5. PEG-Polyester-Derived Thermo-Sensitive Hydrogels

**Figure 4:**
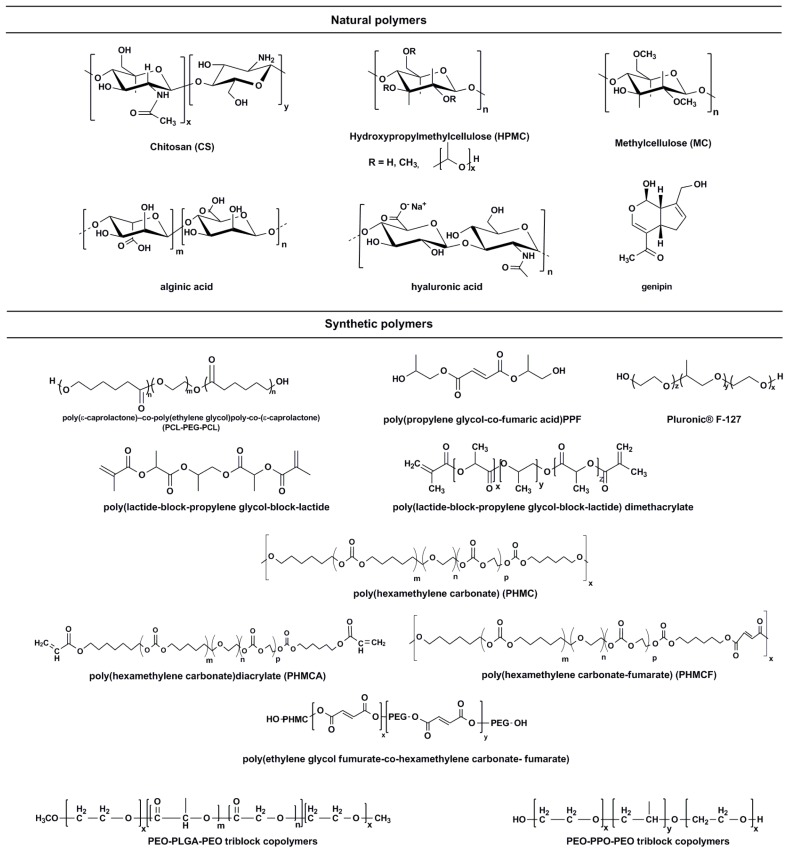
Chemical structures of selected most commonly used thermo-responsive polymers discussed in application for drug and tissue engineering (adapted with permission from Matanovic et al., 2014.

Thermo-sensitive PEG-polyester-based hydrogels include polyglycolide (PGA), polylactide (PLA), poly(δ-valerolactone), polycaprolactone (PCL) and poly(lactide-co-glycolide) (PLGA), which are prepared by linking ends of polyester chains at the PEG ends [117]. The advantages of these polymers is their great biodegradable nature, providing a stimuli responsive and biocompatible platform. Polyester-PEG-polyester structures have been shown to display significant thermo-sensitivity, such as PEG-PLA copolymer, inserting a *p*-dioxanone (DX), resulting in physiological enhanced thermo-responsiveness. According to Kato et al., BMP-loaded PEG-polyester hydrogel was delivered in vivo, using similar polymerization techniques. Results showed a dual responsive pH and thermosensitivity, delivering the bioactive with high loading capacity in the hydrogel, displaying significant regeneration of bone at the site of delivery [118]. According to Shim et al., sulfamethazine oligomer (SMO) was copolymerized with PEG polyester copolymers, reacting to pH and temperature of physiological conditions (pH 7.4 at 37 °C), nonetheless, displaying decreased degradation due to the buffering effects of SMO [119]. Exploring the degree of gelation temperature ranges, it was found that two polyester blocks between the PEG-based polymer, display a wider range of gelation, increasing the mechanical, loading capacity and drug release profiles of the hydrogel system [120]. It was also shown that polyester copolymers such as poly(caprolactone-co-glycolide), poly(lactide-coglycolide) as well as diblock copolymers such as methoxy-terminatedPEG-β-poly(caprolactone-co-lactide) (PCLLA) and methoxy-terminated PEG-PCL (mPEG-PCL) display significant thermo-sensitivity, varying their gelation temperatures over substantial ranges. A drawback of PEG-polyester copolymers are that the by-products are acidic in nature, causing an immune responsive mechanism once injected at the site of delivery [121]. However, these polymers are one of the most frequently used hydrogels for thermo-responsive applications, delivering a diverse range of drugs, proteins and various bioactive molecules, with admirable loading capacity, release rate and biodegradation properties. Figure 4 illustrates the chemical structures of the most commonly used polymers for thermo-responsive tissue and drug engineering applications. Table 1 represents a summary of the various polymers mentioned for tissue engineered applications, as well as their mechanism of gelation, delivering their bio-actives in response to a specific stimulus.

## 4. Injectable/Implantable Research Technologies Employed in Bone Tissue Engineering Applications

### 4.1. Research Focused on Biomedical Applications

Research in the field of bone tissue engineering has gained significant recognition in the last 7 years, with particular attention to polymeric technologies, injection as hydrogels or implantation as scaffolds; 3D printing has also been studied extensively due to the specificity of the conditions used [122]. Much research has focused on bone repair and healing, whilst other areas of research have focused on the regeneration of bone material. In cases of bone injuries and tumor-related defects, these biomedical technologies mimic the mechanical and ideal material composition of bone, delivering a variety of growth factors, biocompatible polymers, proteins, as well as controlled delivery of active pharmaceutical ingredients [123,124,125,126].

#### 4.1.1. Bone Repair

Research in the area of bone repair has demonstrated a vast interest in a variety of strategies to promote osteogenic and angiogenic responses. Of significant interest is the application of collegen-bioglass-derived materials. Various bioglass^®^ forms (45S5, 58S, S53P4) as well as Biosilicate^®^ have gained much popularity in bone repair [127]. Research has also been conducted using collagen-bioactive glass scaffolds, which have demonstrated enhanced mineralization compared to pure collagen-based scaffolds. In addition, researchers further evaluated cell cultured MC3T3-E1 cells in both scaffold variations, demonstrating greater metabolic and alkaline phosphatase (ALP) activity in both scaffolds, with lower swelling in situ when combined with bioglass [128]. Another study reported cobalt-loaded 3D printed macroporous scaffolds of collagen and bioglass, demonstrating a compressive strength in the range of trabecular bone, with significant stability of the scaffold [129]. Studies using chitosan crosslinked with collagen and reinforced with bioactive glass, as a nanoparticulate thermo-responsive injectable system, demonstrated significant gelation at body temperature, with promising potential as an injectable bone tissue engineered system [130]. One of the benefits of injectable treatments compared to implantable technologies is that invasive surgery is kept to a minimum, however implantable scaffolds demonstrated greater immediate weight-bearing, due to the strength and stiffness which is obtained nearly instantaneously from a scaffold implantation [131].

#### 4.1.2. Regeneration of Bone Tissue

In the field of research involving regenerative bone tissue material, various studies have been undertaken, evaluating the differentiation of osteoblasts, various growth factors and re-mineralization in bone tissue. Many studies demonstrated the application of chitosan, crosslinked with various salts and phosphate materials, indicating significant bone regenerative effects. One of the studies using this strategy was conducted by Niranjan and coworkers. They developed a thermo-sensitive hydrogel scaffold of zinc doped chitosan/β-glycerophosphate, which exhibited geometrically defined pore networks, with significant crystalline and swelling properties. The polymeric system was proved to demonstrate antibacterial, biocompatible and substantial osteoblast differentiation [132].

In other research studies, stimuli-responsive polymeric bone regenerative systems involving the use of cyclodextrins, adamantine and crosslinking of PNIPAM was found to be particularly beneficial in bone regeneration applications. A thermo-sensitive hydrogel, crosslinked with the above chemical entities, enabled gelation after injectable delivery in vivo, and was further proved to demonstrate biocompatible and biodegradable properties. Furfurylamine grafted chondroitin sulfate (ChS-F) and maleimido-terminated poly(ethylene glycol) (PEG2K–AMI), significantly increased the mechanical strength of the system, further substantiating that the hydrogel considerably induced bone regeneration when tested in a male Kunming mice model [133].

**Figure 5:**
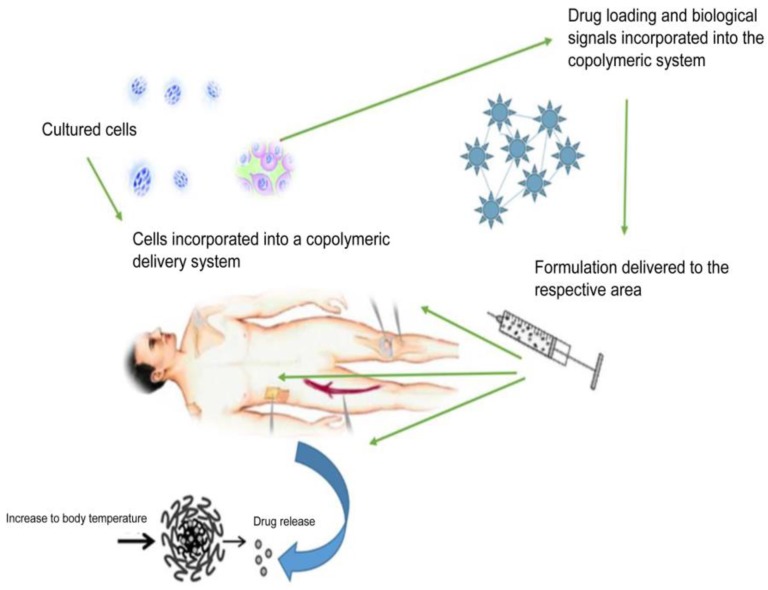
Schematic representation of tissue engineering application, delivering drug-loaded thermo-sensitive polymers with bioactive signals.

Figure 5 outlines the general approach undertaken when formulating a stimuli-responsive bone tissue engineered delivery system, incorporated with biological growth factors and signals, specific cell cultures biocompatible with the copolymeric material, as well as active pharmaceutical ingredients, able to promote bone repair and regeneration. Another study explaining this concept, as seen in Figure 5, successfully proved mineralization, new bone formation and tissue responses to PNiPAAm based hydrogels, evaluated in a rat cranial defected model. The implanted scaffold demonstrated substantial bone regeneration within 12 weeks of implantation. It was reported that the hydrogel promoted matrix hydrophobicity-dependent mineralization, demonstrating significant strategies for stem cell and growth factor biocompatibility within the hydrogel system for effective bone regenerative application [134].

## 5. Nano-Enabled Thermo-Responsive Hydrogel Systems

The integration of stimuli-responsive hydrogels with numerous nanocarriers is a promising technique for extended delivery of growth factors, proteins, polymeric networks of molecules as well as loading of bone repairing and regenerative active pharmaceutical agents. Surface decoration of nanoparticles is also one of the uses of thermo-responsive polymers. The combination of in situ forming gelling systems with nanosized drug delivery, for example diverse liposomes or nanoparticles, have become a topic of growing interest to date, for the strategy of achieving a controlled kinetic system by increasing the surface area to volume ratio of the copolymeric delivery system [135]. The utilization of poloxamer gels and polylactic-co-glycolic acid (PLGA) nanoparticles were investigated in vitro and in vivo. Their combined polymeric properties for subcutaneous delivery of peptides and proteins with short half-lives were also analyzed [136]. Results demonstrated that formulations containing protein-loaded PLGA nanoparticles, possessed greater long-term therapeutic effects, in comparison to non-nano systems evaluated. The incorporation of poly(3-hydroxybutyrate-co-3-hydroxyhexanoate), further exemplified the controlled mechanism of release in the hydrogel, promoting a sustained injectable delivery system, suitable for chronic osteoporosis conditions [137].

With regards to poorly soluble drugs, biodegradable thermo-responsive nanoparticulate hydrogels can be useful for local injectable delivery, especially applicable in small bone fractures, where the use of pins and casts is not practical. To operate as a platform for the slow release of hydrophobic drugs, such as simvastatin, used to promote bone strengthening, thermo-responsive Pluronic F127 hydrogels with poly(ε-caprolactone)-poly(ethylene glycol)-poly(ε-caprolactone) nanoparticles serve as a prototype formulation, among many other crosslinked combinations, having hydrophobic and hydrophilic units, able to load a variety of drugs and BMPs for bone repair and strengthening, releasing them in a controlled mechanistic approach. It was established that the LCST of this system can be improved with a larger mass of the incorporated nanoparticles [138].

Thermo-responsive polymers may also be useful in the biomedical field in the form of nano-carriers [139]. The emergence of several types of nanocarriers with magnetic targeting capability, by coating them with thermo-responsive polymers, have been revealed in numerous articles [140,141,142]. Due to their inimitable properties, these magnetic, redox responsive and thermo-sensitive nanoparticles have demonstrated immense potential in controlled drug delivery and greater therapeutic efficacy in vivo. To establish a targeted drug delivery system in response to higher temperature conditions, novel thermo-responsive nanoparticles formulated by the self-assembly of poly(*N*,*N*-diethylacrylamide-co-acrylamide)-block-poly(γ-benzyl)-l-glutamate were designed and evaluated [143]. It was therefore capable of adjusting the LCST of nanoparticles to a value between body temperature and the temperature in local hyperthermia (~43 °C). This system has significant potential for bone related injuries, especially reacting to the stimulus of inflammatory markers, where greater temperature dependent stimuli-responsive properties in the hydrogel are essential.

## 6. Advancing the Efficacy of Hydrogels in Drug Delivery Applications

Prompt drug release from the gel matrix results in the large water content of nearly all hydrogels, especially in the instance of hydrophobic drugs for which the deliverance of hydrogels is predominantly employed. This release profile is more concise than that which is achieved by macroscopic or microscopic mechanisms. These processes are based on more hydrophobic polymeric systems. Therefore, many different ideas have been investigated to lower the rate of release of drugs within a hydrogel system.

### 6.1. Drug-Hydrogel Interactions

To improve the binding between a loaded drug and the hydrogel matrix, chemical and physical strategies can be utilized to prolong the duration of drug release.

#### 6.1.1. Physical Interactions

To amplify the strength of the interactions between the gel and a target drug, charge interactions between ionic polymers and charged drugs have commonly been utilized to cause the release of the drug to be delayed. Phosphate-functionalized polymers have become useful in this regard. This is particularly due to their multivalent anionic charge. Phosphate-containing soft contact lenses can bind a cationic drug such as naphazoline in amounts which are directly proportional to the phosphate content [144]. Amino functional groups can be used to bring about a delayed release of anionic drugs. Anionic and cationic properties of functional groups can have an influence in extending the release of a drug which is of opposite charge. This is generally found in carbohydrate-based polymers [145]. Hyaluronic acid has been used as a delivery vehicle for local anesthetics due to its charge interactions, biocompatibility and viscosity [146,147,148]. Monomers or polymers with particular nonionic affinities to a specific drug can be copolymerized into hydrogels, as alternatives, to postpone or augment the release of the drug. Amphotericin B, an antifungal drug, has significant interaction with photocross-linked dextran-based hydrogels, which therefore allows contact eradication of fungi for two months [149]. The precise binding mechanism is vague as amphotericin B did not bind to PEG-derived hydrogels of equal chain density, so the precise mechanism of molecular binding remains uncertain.

#### 6.1.2. Covalent Bonding

To ensure that the release of the drug is essentially controlled by the rate of chemical or enzymatic cleavage of the polymer-drug bond, the drug can be covalently conjugated to the hydrogel matrix. For example, to facilitate osteogenic differentiation of human mesenchymal stem cells, dexamethasone has been conjugated to a photo-reactive monoacrylated PEG through a degradable lactide bond [150]. On the other hand, hydrolysis of the polymer backbone can also regulate drug release, perhaps stimulating the release of a relatively altered drug analogue. For instance, methacrylate-functionalized non-steroidal anti-inflammatory drugs have been conjugated to methacrylic functionalized dextrans by means of UV radiation; a chemically modified drug analogue is released as the dextran hydrogel degrades [151,152].

## 7. Analogous Cell and Biomaterial Characterization for In Vitro Success and In Vivo Translation

The efficacy of tissue development should be characterized in vitro and in vivo, after selecting or designing the appropriate biomaterial and cell source for an injectable tissue engineering system. There have been several cell types and polymer systems that have been useful to bone tissue engineering in injectable systems in vivo and in vitro, with regards to cartilage. Paige et al. encapsulated chondrocytes in alginate and thereafter subcutaneously implanted it in mice. This was one of the first examples of injectable cartilage tissue. Matrix production of cartilage-relevant molecules was revealed by histological analysis. In a similar manner, Sims applied chondrocytes to PEG, a synthetic polymer [153]. Cartilage production observed with PEG and alginate was similar. Collagen, PEG encapsulated with poly-lactic or -glycolic groups and polyvinyl alcohol, are other examples of biological and synthetic polymers that have been combined with chondrocytes [154,155]. A clinically used tissue glue, fibrin, could also be combined with chondrocytes to yield high-quality cartilage (as determined through biochemical analysis, as well as histologically) [156,157,158,159]. However, fibrin glue has weak mechanical properties which unfortunately limit its application in cartilage tissue engineering in vivo.

For bone tissue engineering, chondrocytes can be isolated from various cartilages such as costal (rib) [158,160], articular, nasal septum [161,162] as well as auricular [163,164]. Chondrocytes from these sources, in addition to variable ease in clinical access for biopsy, have unpredictable tissue formation and proliferation characteristics. Using chondrocytes isolated from different regions embedded in fibrin glue, cartilage development in an injectable tissue engineering system was evaluated [165]. Considerable differences were observed in mass and biochemical properties of tissue engineered from articular, auricular and costal cartilage, with the greatest size and mechanical properties being generated by auricular-based tissue. Articular chondrocytes were found to have inferior tissue production. This is perhaps due to their requirement for mechanical stimulation which was absent in the subcutaneous environment where the constructs were implanted.

The majority of the injectable cartilage tissue engineering scaffolds rely on hydrogel materials as a scaffold. Mercier et al., in a recent study, obtained a technique to produce an injectable cartilage repair system using a solid polymer, poly(lactide-co-glycolide) (PLG) [166]. PLG was used to create an injectable system by forming microspheres of polymers that were injected with a suspension of chondrocytes.

## 8. Current Research Approaches to Date

Brenden et al., from the Department of Bioengineering of Rice University (Houston, TX, USA) investigated biodegradable, phosphate-containing, dual-gelling macromers for cellular delivery in bone tissue engineering [167]. When elevated to physiological temperature, the injectable, biodegradable, dual-gelling macromers were utilized to encapsulate mesenchymal stem cells within stable hydrogels. To improve the biointegration and to facilitate hydrogel degradation, pedant phosphate groups were incorporated within the *N*-isopropylacrylamide-based macromers. The live cells remained within the hydrogel for 28 days in vitro. Acellular and cell-laden hydrogels were then implanted into a critical-size rat cranial defect for 4 and 12 weeks, and were thereafter shown to degrade in vivo as well as help to facilitate bone growth at the site of defect. Improved bone bridging of the defect was observed, as well as direct bone-to-hydrogel contact was noticed in the majority of the implants. Thus it was concluded that this class of macromers is a promising material for craniofacial bone tissue engineering.

PEG-silk hydrogel combined with polymeric particles was also investigated for delivering rhBMP-2 for bone regeneration [168]. A composite hydrogel was developed which consisted of PLA/PLGA polymeric particles which was used as a BMP-2 delivery vehicle. The initiation of PLA into PEG-silk gels resulted in the hydrogel having new hydrophobicity properties. This inhibited the burst release of BMP-2 as well as improved the gel’s structural ability. Furthermore, these composite gels could stabilize entrapped proteins and conserve their bioactivity in vitro. In vivo, the bio-degradability experiment indicated that this system was biocompatible. The new hydrophobicity properties remarkably decreased the degradation rate. In a rat critical-sized cranial fracture model, the gel containing PLA promoted the most bone formation. The results of this research indicated that the employment of PLA changed the physicochemical properties of gels more appropriate as a BMP-2 carrier and this induced bone regeneration significantly in large bone fractures.

Biomineralization of calcium phosphate crystals on chitin nanofiber hydrogels for bone regeneration material was investigated by Mari and co-workers [169]. A calcium phosphate/chitan nanofibre hydrogel was prepared for bone tissue engineering. Calcium phosphate was mineralized on the hydrogel. This was done by incubation in a solution of diammonium hydrogen phosphate followed by calcium nitrate tetrahydrate. The formation of calcium phosphate crystals was revealed by Fourier transform infrared spectroscopy (FTIR) and X-ray diffractometry (XDR). The morphology of the calcium phosphate crystals was contingent on the calcification time. The reinforcement effect of the calcium phosphate crystal lead to the improvement of the mechanical properties of the hydrogel after mineralization. Calcium phosphate/chitin nanofibre hydrogel increased mineralization in subcutaneous tissues in an animal model. Morphological osteoblasts were also noticed.

Studies using hyaluronic acid (HA)-based hydrogel scaffolds with a triple degradation behavior for bone tissue engineering was also strategically investigated [170]. HA hydrogels which have a triple degradation behavior were synthesized to better mimic the nature of the bone extracellular matrix. These hydrogels were synthesized from 3,3′-dithiodipropionate hydrazide-modified HA (DTPH–HA) and polyethylene glycol dilevulinate (LEV–PEG–LEV) through the reaction of ketone carbonyl groups of LEV–PEG–LEV with the hydrazide groups of DTPH–HA. Results from the study revealed that the HA hydrogels displayed a very porous morphology and had pore diameters which ranged from 20 to 200 μm. Hyaluronidase and reducing substances or acidic pH values could be used to degrade the HA hydrogels. Osteoblast-like MC3T3-E1 cells by live/dead staining and MTT assays were used to analyze the biocompatibility of the HA hydrogels. The results indicated that these hydrogels had very good compatibility and could also support the attachment and proliferation of MC3T3-E1 cell. All the results of this experiment revealed that the HA hydrogels synthesized by hydrazone bond crosslinking have substantial potential to be used in bone tissue engineering.

It can thus be concluded that all the above mentioned studies are promising research avenues in the field of bone tissue engineering. Many of these studies have been done more than one research platform, confirming similar positive correlation results. These responsive polymers are significantly capable of promoting new cell growth and increasing the rate of tissue repair. Furthermore, these studies have been evaluated in vivo, producing significant responses to the bioengineered system, having a defined positive potential advancement for promoting bone regeneration and repair.

## 9. Future Research Perspectives in the Field of Bone Tissue Engineering and Drug Delivery

A great emphasis has been given to the development of tough and durable scaffolds which will be able to sustain loading in vivo for moderate and high load-bearing applications, although the mechanical design requirements for bioresorbable scaffolds differ considerably depending on the functional requirements of the bone it is replacing. Natural bone achieves its distinctive amalgamation of mechanical properties from an architectural design that extends from nanoscale to macroscopic dimensions, with accurately and carefully engineered interfaces. The bone’s fracture resistance stems from toughening mechanisms at these dimensions. The smallest length-scales determine the bone’s intrinsic fracture resistance by encouraging plasticity as well as impacting on bone strength. The larger-scales extrinsically strengthen bone by protecting the growing crack. Thus far, there are no synthetic biomaterials with such a structure. However, these unique mechanical properties could be achieved through combining mechanisms acting at various length scales.

Much has been done to manipulate the mechanical properties, such as strength, toughness and stiffness, of scaffolds through inventing hydrogels and nanostructures, e.g., nanoparticles or nanofibre reinforcements in polymer matrices, to imitate the bone’s natural makeup. It was established that synthetic whitlockite (WH: Ca_18_Mg_2_(HPO_4_)_2_(PO_4_)_12_) nanoparticles can recapitulate early-stage of bone regeneration. This can be achieved by prohibiting osteoclast activity, stimulating osteogenic differentiation, and transforming into mechanically enhanced hydroxyapatite (HAP)-neo bone tissues by the continuous supply of PO_34_ and Mg_2_þ under physiological conditions. Based on structural analysis, the dynamic phase transformation from WH to HAP contributed as a crucial factor for bone regeneration [171]. Nevertheless, it was noted that although the incorporation of hydrogel and nanoscale reinforcement increases the strength, the toughness drops drastically. Therefore, the formulation of stronger and tougher scaffold constituents entails the combination of a hierarchical design incorporating many length-scales to produce strength, i.e., to mimic composite deformation of hydrogels and nanocrystals of hyaluronic acid and collagen, together with the micro-level structures to produce toughness, e.g., to mimic osteons and cement lines.

Hydrogels and scaffolds could play a more active role in the regeneration process of bone, which need many biological events in which growth factors supply signals to initiate healing. Bone development in vivo particularly involves the release of chemicals, such as growth factors, at crucial time points to trigger osteoinduction and thus bone regeneration could be markedly increased by the confined delivery of suitable growth factors, such as TGF-β, BMPs, IGF, or FGF. Drug release spatio-temporal profiles could be formulated to resemble signaling in vivo if the signaling for osteoinduction could be specified. The four fundamental questions that should direct the design of chemical release tools are: what to release, when, where and how much [10].

Another drug delivery alternative is the utilization of polymer-based scaffolds with various chemicals contained within the polymer. The use of glass-based scaffolds, e.g., Bioglass^®^, for sustained release of ions is an appealing modification of this approach. Bioactive glass can chemically bond to bone, whilst degrading in the body in a very controlled fashion. Many bioactive glasses have been formulated with various degradation rates while targeting multiple applications, as the glass composition can be effortlessly altered and several different ions could be integrated. Examples of these include implant coatings, scaffold fabrication and bone fillers. The release of Si from bioactive glasses could be advantageous. The release of other ions from the glass in vivo could be employed to assist in angiogenesis, e.g., Cu^2+^, as well as differentiation towards osteoblastic lineages, e.g., Sr. Various glass constituents could be used to manipulate the release rates.

Research was carried out on novel polyvinyl alcohol-bioglass 45S5-based composite nanofibrous membranes as bone scaffolds [172]. Composite nanofibrous membranes based on sol-gel derived 45 SiO_2_ 24.5 CaO 24.5 Na_2_O 6 P_2_O_5_ (bioglass, BG) and 43 SiO_2_ 24.5 CaO 24.5 Na_2_O 6 P_2_O_5_ 2 Fe_2_O_3_ (magnetic bioglass, MBG) blended with polyvinyl alcohol (PVA) have been electrospun. These membranes were amorphous in structure with negligible crystalline precipitates. These membranes possessed a biodegradable nature. The composites displayed increased tensile strength, greater proliferation of human osteosarcoma MG63 cells and increased alkaline phosphatase enzyme activity than the bare PVA membrane. It was thus proved that these composites possessed substantial potential in bone tissue engineering. Nonetheless, the effect of pH changes prompted by the degradation of glass, the ideal release rates, as well as the role of glass surface roughness should be studied systematically.

In summary, new design concepts and formulation approaches are desperately required to produce novel scaffolds for bone regeneration and repair. Specifically, more research is needed in the field of bone tissue engineering to discover the relationships that connect composition and material architecture at multiple-length scales with macroscopic mechanical behavior and the potential for osteogenesis. The results of this research could be utilized to fabricate new systems that have the capability to manipulate chemistry and cellular responses. This knowledge will also serve to conduct systematic studies on surface roughness and drug delivery profiles. The great possibilities opened by the increasing emphasis on hydrogels and nanotechnology in the field of bone tissue engineering now permit the modification of scaffold chemistry with a revolutionary degree of control. The physicochemical environment could be monitored and manipulated to observe the key cellular events at the molecular level. The results of such bone tissue engineering could create an ideal bone scaffold that could be used in the treatment of bone defects. The goal is to produce “active” scaffolds that are explicitly fabricated for bone regeneration that will provisionally substitute for natural tissues, while interacting with their surroundings, responding to environmental changes, as well as actively direct cellular events. These scaffolds will combine with bone tissue while they are actively absorbed, with controlled osteogenic activity, which will take advantage of the biological principles of bone repair and regeneration. As a result, these capabilities will produce faster bone formation, increased healing time and speedy recovery to function.

## 10. Conclusions

Injectable stimuli responsive hydrogels exhibit a significant platform for bone tissue engineering with the benefit how cells as well as drugs can be infused into the gelling matrix. The success of injectable tissue constructs is greatly dependent on chemical, physical and biological properties. Hydrogels display water based homogenous properties to encapsulate, manipulate and transfer its contents to the surrounding tissue, in the least invasive manner. Numerous attempts have been made to augment injectable hydrogels, and accordingly, assist the enhancement of natural and functional tissues. Thus far, injectable tissue engineering in the field of orthopedic sciences has produced countless benefits over previous application methods. Irrespective of shape and defect geometry, injectable therapy has an unparalleled advantage in which intricate sites of therapy can be effortlessly targeted with minimum invasive procedures. The field of theremoresponsive hydrogels is of great interest to biomedical researchers and engineers. Excellent progress has been made by studying the different natural and synthetic polymers that can be utilized in this field of bone tissue engineering and thus novel materials have been proposed. Key factors such hydrophilic/hydrophobic balance in the molecular composition have been modified in order to produce hydrogels with desired features. Biodegradability, physiological range, as well as augmentation of mechanical properties have been an important research focus, producing exciting potential for employing thermoresponsive hydrogels in bone tissue engineering applications. The global bone graft substitutes market is growing rapidly due to patient need and health care improvement. The design and production of biodegradable hydrogels for bone tissue engineering applications has thus become a major research and development interest, with significant bone tissue technologies currently being investigated to date, thus expecting major advancements in the near future for bone tissue engineered delivery systems.

## Figures and Tables

**Table 1:** Summary of injectable hydrogels for tissue engineering.

Hydrogel Source	Polymer/s Used	Gelation Mechanism	References
Natural	Hyaluronic acid, chitosan	Thermal/chemical/free radical crosslinking	[59,65,66,68]
	Cellulose, Agarose, Matrigel	Thermal crosslinking	[75,76,78]
	Gelatin, Collagen	Thermal/chemical crosslinking	[81,82,83]
Synthetic	PDMA, PHPMA, PNVCL		
	PNIPAAm, Fibroin, PLA		
	NIPAAm/MBAAm		
	Oligolactide-(2-HEMA)	Thermal crosslinking	[87,88,91,92],
	OligoLA-HEMA, L-Lysine		[97,98]
	PNIPAAm-Co-HEMA)		
	P(L-glutamic acid)		
	POEGMA, OEGMA, MEO2MA	ATRP crosslinking	[101,102,103]
	Poly(organophosphazene)		
	a-amino-w-methoxypoly(ethylene glycol), lleoEt	Chemical crosslinking	[104,106,108]
	PEO-PPO-PEO, mPEG-PCL		
	PEG, PGA, PLA, PCL, PLGA	Thermal crosslinking	[111,112,114],
	PVL, DX, SMO, PCLLA		[117]
